# Supplementary material for: Environmental Safety Assessments of Lipid Nanoparticles Loaded with Lambda-Cyhalothrin
Source: Nanomaterials (Basel). 2022 Jul 27;12(15):2576. doi: 10.3390/nano12152576 (PMC9370418; doi:10.3390/nano12152576)
Supplement: Supplementary file 1 [file nanomaterials-12-02576-s001.zip › nanomaterials-1804613-supplementary.pdf]

nanomaterials

Supplementary Materials

Environmental safety assessments of lipid nanoparticles loaded with lambda-cyhalothrin

Catarina Ganilho [1], Márcia Bessa da Silva [1], Cristiana Paiva [1], Thacilla Ingrid de Menezes [2], Mayara Roncaglia dos Santos [2], Carlos M. Pereira [2], Ruth Pereira [1,*] and Tatiana Andreani [1,2,3,*]

[1] GreenUPorto, Sustainable Agrifood Production Research Centre & INOV4AGRO, Department of Biology, Faculty of Sciences, University of Porto, Rua Campo Alegre s/n, 4169-007 Porto, Portugal; up201707255@edu.fc.up.pt (C.G.); bessamiss@gmail.com (M.B.d.S.); cristiana.pgpaiva@fc.up.pt (C.P.)
[2] Chemistry Research Centre (CIQUP) & Institute of Molecular Sciences (IMS), Department of Chemistry and Biochemistry, Faculty of Sciences, University of Porto, Rua do Campo Alegre s/n, 4169-007 Porto, Portugal; up201811986@edu.fc.up.pt (T.I.d.M.); mayara.santos@fc.up.pt (M.R.d.S.); cmpereir@fc.up.pt (C.M.P.)
[3] Centre for Research and Technology of Agro-Environmental and Biological Sciences (CTAB) & INOV4AGRO, University of Trás-os-Montes e Alto Douro, UTAD, 5000-801 Vila Real, Portugal
* Correspondence: ruth.pereira@fc.up.pt (R.P.); tatiana.andreani@fc.up.pt (T.A.); Tel.: +351-220-402-000 (R.P. and T.A.)

Dehydrogenase

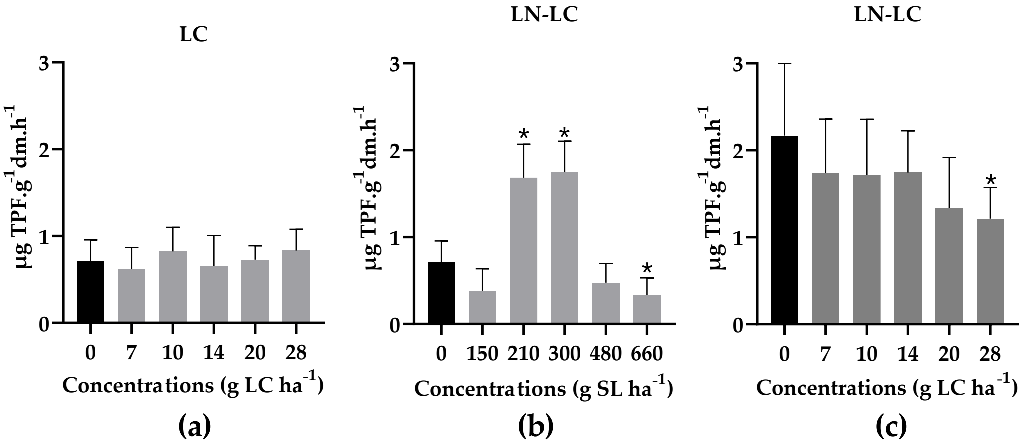

Figure S1. Average dehydrogenases activity in soils exposed for 15 days to different concentrations of LC (**a**), LN (**b**) and LN-LC (**c**). The concentrations tested are based on the amount of the LC. In the case of LN, the concentrations indicated are of solid lipid (SL) used in the synthesis of LN. The same concentrations of SL were tested for LN-LC. Results are represented by mean ± SD values. The asterisks mark the significant differences from the control group (0 g LC ha^{-1}) ($p < 0.05$, Dunnett).

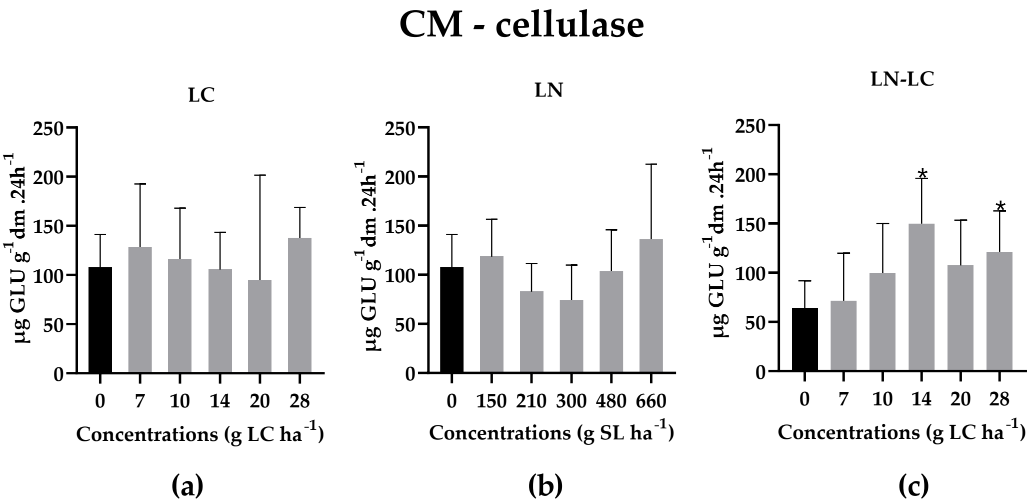

Figure S2. CM–cellulase activity in soils exposed for 15 days to different concentrations of LC (**a**), LN (**b**) and LN–LC (**c**). The concentrations tested are based on the amount of LC. In the case of LN, the concentrations indicated are of solid lipid (SL) used in the synthesis of LN. The same concentrations of SL were tested for LN–LC. Results are represented by mean ± SD values. Asterisks mark the significant differences in relation to the control group (0 g LC ha^{-1}) ($p < 0.05$, Dunnett).

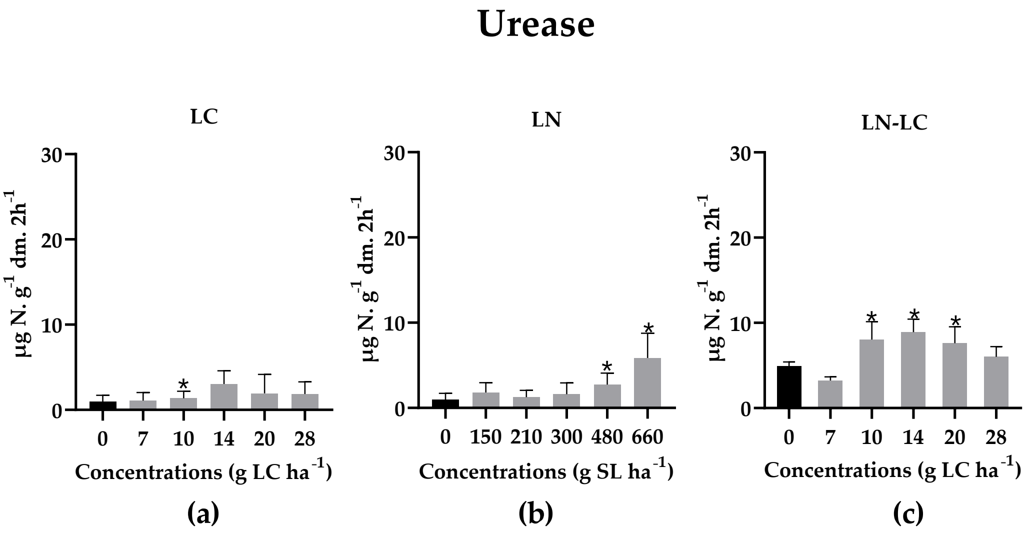

Figure S3. Urease activity in soils exposed for 15 days to different concentrations of LC (**a**), LN (**b**) and LN–LC (**c**). The concentrations tested are based on the amount of LC. In the case of LN, the concentrations indicated are of solid lipid (SL) used in the synthesis of LN. The same concentrations of SL were tested for LN–LC. Results are represented by mean ± SD values. Asterisks mark the significant differences in relation to the control group (0 g LC ha^{-1}) ($p < 0.05$, Dunn).

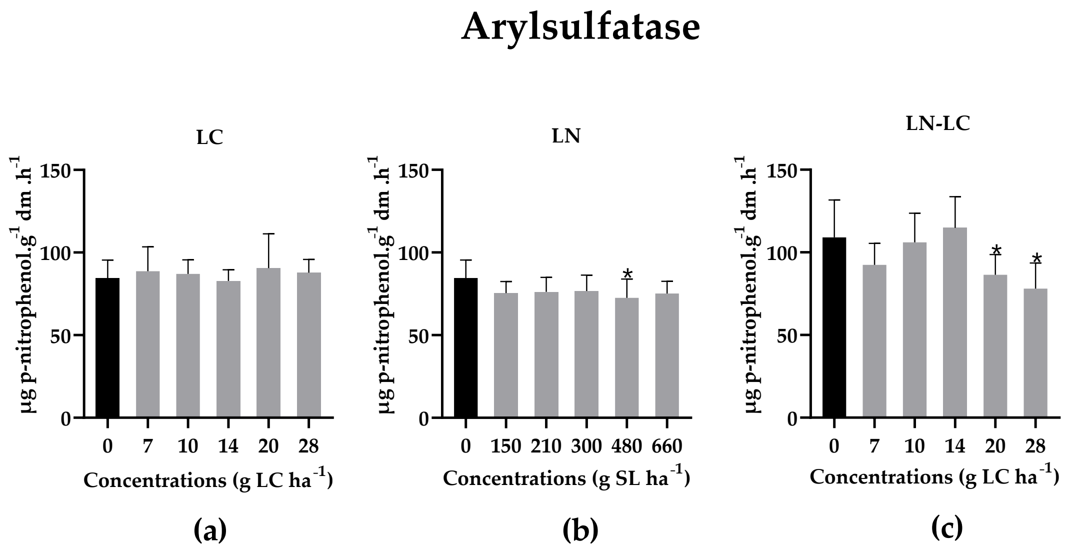

Figure S4. Arylsulfatase activity in soils exposed for 15 days to different concentrations of LC (**a**), LN (**b**) and LN–LC (**c**). The concentrations tested are based on the amount of LC. In the case of LN, the concentrations indicated are of solid lipid (SL) used in the synthesis of LN. The same concentrations of SL were tested for LN–LC. Results are represented by mean ± SD values. Asterisks mark the significant differences in relation to the control group (0 g LC ha^{-1}) ($p < 0.05$, Dunnett).

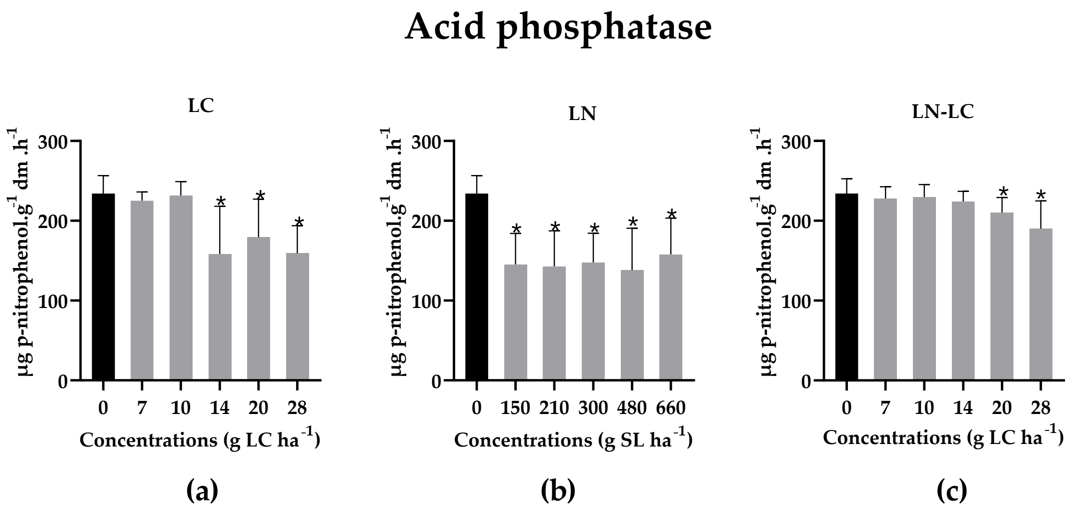

Figure S5. Soil acid phosphatase acid enzymes activity in soils exposed for 15 days to different concentrations of LC (**a**), LN (**b**) and LN–LC (**c**). The concentrations tested are based on the amount of LC. In the case of LN, the concentrations indicated are of solid lipid (SL) used in the synthesis of LN. The same concentrations of SL were tested for LN–LC. Results are represented by mean ± SD values. Asterisks mark the significant differences in relation to the control group (0 g LC ha^{-1}) ($p < 0.05$, Dunn).

© 2022 by the authors. Licensee MDPI, Basel, Switzerland. This article is an open access article distributed under the terms and conditions of the Creative Commons Attribution (CC BY) license (http://creativecommons.org/licenses/by/4.0/).
